# The petrous bone contains high concentrations of osteocytes: One possible reason why ancient DNA is better preserved in this bone

**DOI:** 10.1371/journal.pone.0269348

**Published:** 2022-10-25

**Authors:** Jamal Ibrahim, Vlad Brumfeld, Yoseph Addadi, Sarah Rubin, Steve Weiner, Elisabetta Boaretto

**Affiliations:** 1 Scientific Archaeology Unit, Weizmann Institute of Science, Rehovot, Israel; 2 Department of Chemical Research Support, Weizmann Institute of Science, Rehovot, Israel; 3 Department of Life Sciences Core Facilities, Weizmann Institute of Science, Rehovot, Israel; 4 Department of Molecular Genetics, Weizmann Institute of Science, Rehovot, Israel; 5 Department of Chemical and Structural Biology, Weizmann Institute of Science, Rehovot, Israel; Griffith University, AUSTRALIA

## Abstract

The characterization of ancient DNA in fossil bones is providing invaluable information on the genetics of past human and other animal populations. These studies have been aided enormously by the discovery that ancient DNA is relatively well preserved in the petrous bone compared to most other bones. The reasons for this better preservation are however not well understood. Here we examine the hypothesis that one reason for better DNA preservation in the petrous bone is that fresh petrous bone contains more DNA than other bones. We therefore determined the concentrations of osteocyte cells occluded inside lacunae within the petrous bone and compared these concentrations to other bones from the domestic pig using high resolution microCT. We show that the concentrations of osteocyte lacunae in the inner layer of the pig petrous bone adjacent to the otic chamber are about three times higher (around 95,000 lacunae per mm^3^) than in the mastoid of the temporal bone (around 28,000 lacunae per mm^3^), as well as the cortical bone of the femur (around 27,000 lacunae per mm^3^). The sizes and shapes of the lacuna in the inner layer of the petrous bone are similar to those in the femur. We also show that the pig petrous bone lacunae do contain osteocytes using a histological stain for DNA. We therefore confirm and significantly expand upon previous observations of osteocytic lacuna concentrations in the petrous bone, supporting the notion that one possible reason for better preservation of ancient DNA in the petrous bone is that this bone initially contains at least three times more DNA than other bones. Thus during diagenesis more DNA is likely to be preserved in the petrous bone compared to other bones.

## Introduction

The possibility of extracting genetic information from fossil material was first demonstrated by Higuchi et al. [1]. Many subsequent studies have confirmed the presence of ancient DNA in fossil bones [2–5], but have also shown that contamination and preservation are severe problems [6, 7]. In 2014 Gamba et al. made a remarkable discovery: aDNA is better preserved in the petrous bone that surrounds the otic chamber as compared to other bones [8]. This has been confirmed by other studies on fossil petrous bones [9–11]. The results obtained from analyzing aDNA in fossil petrous bones has and is contributing significantly to our understanding of ancient genomes, and in particular those of hominins. For example, successful retrieval of aDNA in the petrous bone was carried out for the European Late Upper Paleolithic around 13.3k years ago from western Georgia and 13.7k years ago from Switzerland, and from a Mesolithic male from western Georgia 9.7k years ago [12]. A study of 230 ancient Eurasians who lived between 6500-300BC was made possible by obtaining excellent aDNA amounts from petrous bones [13]. Genome-wide data extracted from 16 prehistoric petrous bones of Africans hominins unraveled episodes of natural selection in southern African populations [14]. However, the reasons why DNA is better preserved in the petrous bone compared to other bones are not known. It has often been assumed that this better preservation is somehow related to the unusually high density of the petrous bone [8]. Other hypotheses for better DNA preservation are the presence of a large number of mineralized osteocytes may facilitate the preservation of DNA in fossil bones [15], that the petrous bone is essentially not remodeled [16], or bones that are most likely to preserve DNA are those with the highest amounts of endogenous DNA in vivo, namely bones with the highest concentrations of osteocytes [17].

Our study focuses on the part of the petrous bone that is usually referred to as the otic capsule [18]. This is the part that immediately surrounds the inner ear spaces. The otic capsule consists of 3 layers of bone. From the outside inward, the periosteal layer, the endochondral layer and the inner endosteal layer [18]. The human petrous bone embryology, structure and extreme hardness were studied for more than a century [19]. Mineralization and structural differences in the layers of the petrous bone are documented based on microradiography and circularly polarized light [20]. Doden and Halves showed that the inner layers are more mineralized than the outer layer and the endochondral layer contains localized discontinuous regions that are hypermineralized [20]. In a series of studies on quantum bone remodeling in young pigs and other species, Sorensen et al. showed that remodeling in the otic capsule and its surrounding bone increases with increasing distance from the otic chamber [21–24].

Bloch et al. studied viable and non-viable osteocytes in a 12-mm wide perilabyrinthine bone zone, and tested 65 bulk-stained undecalcified human temporal bones and 19 ribs and reports on viability of cells and shows high numbers of viable osteocytes [25]. The osteocyte lacunae densities in the inner and outer layers of the human petrous bone range between 75–90,000 osteocytes per mm^3^ and 50–61,000 osteocytes per mm^3^ respectively [25]. Osteocyte lacunae have also been quantified in human archaeological bones [26, 27]. Bloch et al. reported on the spatial distribution of osteocytes and in the human labyrinthine capsule [28]. High rates of osteocyte necrosis in the endochondral layer were reported [25]. Bulk staining shows that lacunae and canaliculi seal off the apoptotic DNA contents which may be another explanation for better preservation of DNA [28]. Here we address the hypothesis that one possible reason that DNA is relatively well preserved in fossil petrous bone is because the fresh petrous bone contains significantly more DNA than other bones [15]. Andranowski measured DNA yields from different bone tissues (excluding the petrous bone) and assumed a correlation between the number of osteocyte lacuna numbers and DNA yields. However Andronowski et al. concluded that residual soft tissue likely contributed to the higher DNA yields but not lacunae numbers [29]. In this study we quantify the concentration of bone cell cavities, namely osteocyte lacunae (OL) in pigs. We show that there are up to 3 times more lacunae in the inner layer of the petrous bone compared to the mastoid of the temporal bone and the femur, confirming observations in human petrous bone [25, 27]. These petrous bone osteocytes are therefore likely to be a concentrated source of DNA embedded in bone and hence this could be one explanation for improved preservation of aDNA in the petrous bone.

## Materials and methods

### Materials

#### Osteocyte lacuna counting

Freshly sacrificed adult pigs (*Sus scrofa*) 7 months old (L2) and 5 months old (L3) were supplied by Lahav Clinical Research Organization C.R.O. (Israel). Samples extracted from the skulls included petrous bones, part of the temporal bone which was analyzed from two regions (layers) based on the degree of calcification: outer layer samples (>3mm from the otic cavity (OP2 from skull L2 and OP3 from skull L3)), and inner layers samples (<1mm from otic cavity (IP2 and IP3)). From the same skulls two samples were obtained from the mastoid part of the temporal bone (T2 and T3). Two cortical bone samples from the proximal cortex of the femora of two different adult pigs (F1 and F2; 7 months old supplied by Lahav C.R.O) that were stored in a freezer for 6 years. All cleaned samples were stored in a -20°C freezer prior to analysis.

#### Nuclei staining

The left petrous and mastoid bones were extracted from pig L3 (5 months old) obtained from Lahav C.R.A. (Israel). In addition, six petrous bones were extracted from 3 freshly sacrificed wild-type (ICR CD-1) female mice. The mice experiments were pre-approved by the Institutional Animal Care and Use Committee (IACUC) of the Weizmann Institute. All reagents for bone clearing were purchased from Sigma Aldrich, and DRAQ5 for nuclear DNA staining was purchased from eBioscience™ (cat. No. 65-0880-92).

### Methods

**Fig 1:**
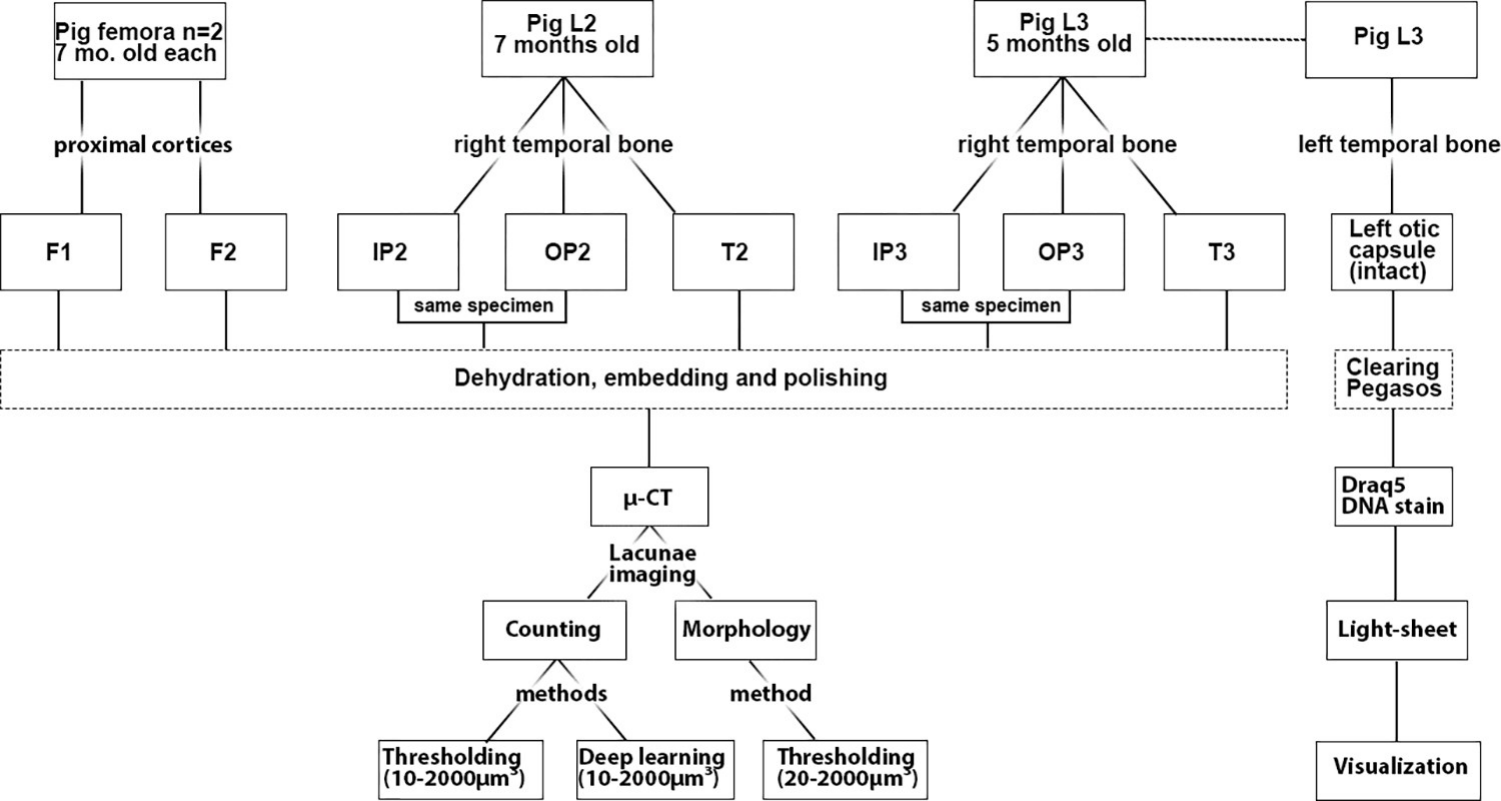
Flow chart of the pig samples in this study. The animal used is indicated at the top row, and the samples extracted from each animal are indicated in the second row. The third row shows sample preparation and then the flow of analyses after each experiment.

Fig 1 is a flow chart showing the samples, as well as the applied methods and analyses.

### Characterizing lacunae concentration and morphology

#### Sample preparation

The two pig skulls (L2 and L3) were dissected and cleaned immediately after sacrifice. All C.R.O. research studies are coordinated and approved by the national ethics committee for animal studies in Israel. All facilities and activities of Lahav C.R.O. are accredited in accordance with GLP and ISO9001 (2015). Cutting and isolation of the skull bone using a manual saw started 3 days after storing at 4°C. For each skull, a sagittal cut was first made at the center of the lateral end of the skull, halving the occipital protuberance and separating the occipital condyles. The cut continued until it reached the parietal bone dorsally and the palatine bone ventrally. Then second and third sagittal cuts were made on each side of the skulls between the zygomatic arch and the external acoustic opening at an angle of 50° towards the parietal bone dorsally and the palatine bone ventrally. Each part of the skull that was extracted included one petrous bone, part of the parietal bone, foramen magnum, the foramen orbitorotundum, the bony tentorium cerebelli, the cranial cavity, one side of the paracondylar process and one side of the tympanic bulla (S2 Movie in S1 File). After isolation of the bones, all soft tissues were discarded, and the bones were washed with double distilled (DD) water.

Using a surgical blade, the meninges was cut with the help of forceps to enable release of the petrous bone. Then the skull piece was mounted on a vice and the temporal bone was forced wide open in the sagittal direction to release the petrous bone part, without causing any deformation or breakage of the petrous bones. The cortical bones of two pig femora were sampled from the cortex of proximal extremities.

All bone samples were washed three times in PBS solution with gentle shaking for 30 min each time. Then the bones were fixed overnight with 3% PFA, and the next day the bones were lyophilized overnight. After dehydration, the bone samples were then embedded in Epon (VersoCit- 2kit, Struers, USA) and allowed to polymerize at room temperature for about 15 minutes. The embedded samples were then cut precisely using a water-cooled diamond disk (Isomet, Buehler Ltd. USA). A transverse cut with slight tilting was made at the preferred location to include all layers of the pig petrous bone [21]. The petrous bone samples were cut in the transverse plane to include the vestibular region and some cross-sections of the semicircular canals. The inferior sections of the petrous bones were used in this study. The cut section surfaces were then ground manually starting with grid 350 paper for 3 minutes and increasing gradually to grid 600 paper for 3 minutes each. The sections were then polished using a 1μm diamond suspension for 5 minutes. All polished sections were imaged using a light microscope in reflected light (Nikon Eclipse, USA).

#### SEM imaging

The polished surfaces were then imaged using a back scattered electron detector in a Phenom SEM (Phenom World, USA). Tiles of images were obtained automatically at 1500x magnification and then automatically stitched together in automated image map (AIM).

#### Micro CT imaging

At first, low resolution (voxel size 12 μm) CT scans were obtained and analyzed to enable selection of regions of interest for the high resolution scans. Then high resolution micro-CT scans were obtained with a small voxel size to detect and count osteocyte lacunae within small volumes of bone. The high resolution μ-CT scans were performed under the same scanning conditions: objective 0.4x, voltage = 60kV, power = 5W. Automatically reconstructed volumes had an average bone volume of 0.4±0.1mm^-3^, and voxel size of 0.85±0.1μm (n = 8). This should be sufficiently small to accurately estimate OL volumes. All high resolution μ-CT scans were imaged over 360 degrees using 1601 projections. We do note however that the two samples of the mastoid bone were embedded in large volumes of plastic. This resulted in a reduction in the signal intensity, and hence an increase in the noise of the data.

#### Lacunae counting

We used two methods for image segmentation. Firstly, we used the FIJI (NIH, USA) software for traditional segmentation by applying the mean thresholding function for reproducibility [30]. The same threshold was used for all samples. Secondly, we segmented the images by applying a deep learning model (DLM) for image segmentation. Each μ-CT reconstructed volume had a specially trained model. For this we used the Dragonfly image analysis and deep-learning software (version 2022.1 build 1249, Objects Research Systems, Montreal, QC, Canada). For the DLM we used the Snap Tool for manually segmenting the OL and canals. Everything else is considered background. To train the DLM, two frames (slices or sections of slices) were selected randomly and segmented manually. These two frames are used as training input for the convolutional neural network (CNN) using the default CNN architecture: U-net with a depth of 5 layers and 64 convolutional filters per layer. After the first DL model completed the training, predictions are generated on new frames and corrections are applied on these frames when needed. These corrections add more information to the second and final CNN training for the model [31]. The final model trained for an additional 100 epochs. The “early stopping” option was selected while training, that is, if the model is not improving for 10 consecutive epochs the training is aborted to review the training results. In all of the scans for both models it took two training sessions until satisfactory and accurate segmentation of the three labels was achieved.

Thresholded datasets using the mean threshold (MT) were loaded onto Dragonfly software for 3D image analysis using connectivity. Connected component refers to a set of voxels that are connected to each other in 3D reconstructions. Connected component labeling marks each connected component with a distinctive label. For both segmentation methods (MT and DLM) we extracted labels using the 6-connected components analysis function and then digitally obtained only labels with volumes between 10 and 2000 μm^3^, because objects smaller than 10 μm^3^ are at the resolution limit of the scan and are therefore subjected to significant errors. Objects larger than 2000 μm^3^ are channels and cavities not related to lacunae. See reference [32]. Counting of segmented objects (known as ‘labels’) is done by the software. Each label or set of labels can be analyzed independently for volume, aspect ratio, distance from a given point, etc. For OL concentrations we analyzed two duplicates derived from two different animals for each of IP, OP, T and F samples. OL concentration was defined as the number of segmented labels in 1mm^3^.

#### Lacunae morphometric analysis

For morphological analysis of OL we used (MT) segmented labels with volumes in the range of 20–2000 μm^3^. For morphometric analysis we obtained the volume and the aspect ratio of the OL labels directly from the label analysis option in Dragonfly, and the aspect ratio ψA (0 < ψA≤ 1) was determined by dividing the minimum orthogonal Feret diameter and maximum Feret diameter. The aspect ratio provides a good indication of the extent of elongation of the lacunae.

### Staining of cell nuclei

#### Bone clearing

After extraction of the petrous bones, all samples were washed with phosphate buffered saline (PBS) three times for 30 min each, then fixed in 4% paraformaldehyde (PFA) overnight. Bone clearing was carried out using the PEGASOS method [33]. Briefly, bone samples were immersed in 20% EDTA (pH 7.0) at 37°C in a shaker for 4 days for the mouse petrous bone and 16 days for the pig petrous bone (L3) until complete decalcification was achieved. The mice experiments were pre-approved by the Institutional Animal Care and Use Committee (IACUC) of the Weizmann Institute. To remove excess EDTA, samples were then washed with distilled water for 30 min. Then, decolorization with 25% w/v Quadrol (Sigma-Aldrich; 122262) for 2 days at 37°C in a shaker. Samples were then placed in tert-butanol (tB) (Sigma-Aldrich; 471712) and 3% w/v Quadrol for delipidation for 2 days with gradual increasing of (tB) concentration (30%, 50% and 70% v/v). Then 70% v/v tert-butanol with 30% v/v polyethylene glycol (Sigma-Aldrich; 447943) (tB-PEG) and 3% w/v Quadrol solution was used for 2 days for dehydration. Samples were then immersed in 75% v/v benzyl-benzoate (Sigma-Aldrich; B6630) with 25% v/v polyethylene glycol (BB-PEG) medium at 37°C for at least 1 day for clearing. All samples were preserved in freshly prepared BB-PEG clearing medium (refractive index R.I. = 1.54) at room temperature in the dark.

#### Nuclei staining

Samples were submerged in a DRAQ5 solution dissolved in PBS (1:1500) for two days with gentle shaking, in order to stain the osteocyte lacunae nuclei, and stained samples were then placed back into BB-PEG clearing medium [34, 35].

3D images of cleared bone were acquired using a light-sheet microscope (Ultramicroscope II, LaVision Biotec) operated by the ImspectorPro software (LaVision BioTec). The microscope is equipped with an Andor Neo sCMOS camera (2,560 × 2,160, pixel size 6.5 μm x 6.5 μm) 16 bit, and an infinity corrected setup 4X objective lens: LVBT 4X UM2-BG (LVMI-Fluor 4X/0.3 Mag. 4x; NA: 0.3; WD: 5.6–6.0 mm), with an adjustable refractive index collar set to the refractive index of BB-PEG (1.54). The light sheet was generated by scanning a supercontinuum white light laser (emission 460 nm– 800 nm, 1 mW/nm– 3 (NKT photonics). The following excitation band pass filters were used: 470/40 nm for tissue autofluorescence–providing general morphology. 617/83nm for DRAQ5. The light sheet was used at 80% width and maximum NA (0.154). Laser power of 80%. The emission filters used were: 525/50 for autofluorescence, 690/50 for DRAQ5. Stacks were acquired using 5 μm step-size and a 200ms exposure time per step. Imaris (Bitplane) was used to create 3D reconstructions and animations of the bone.

### Results

**Fig 2:**
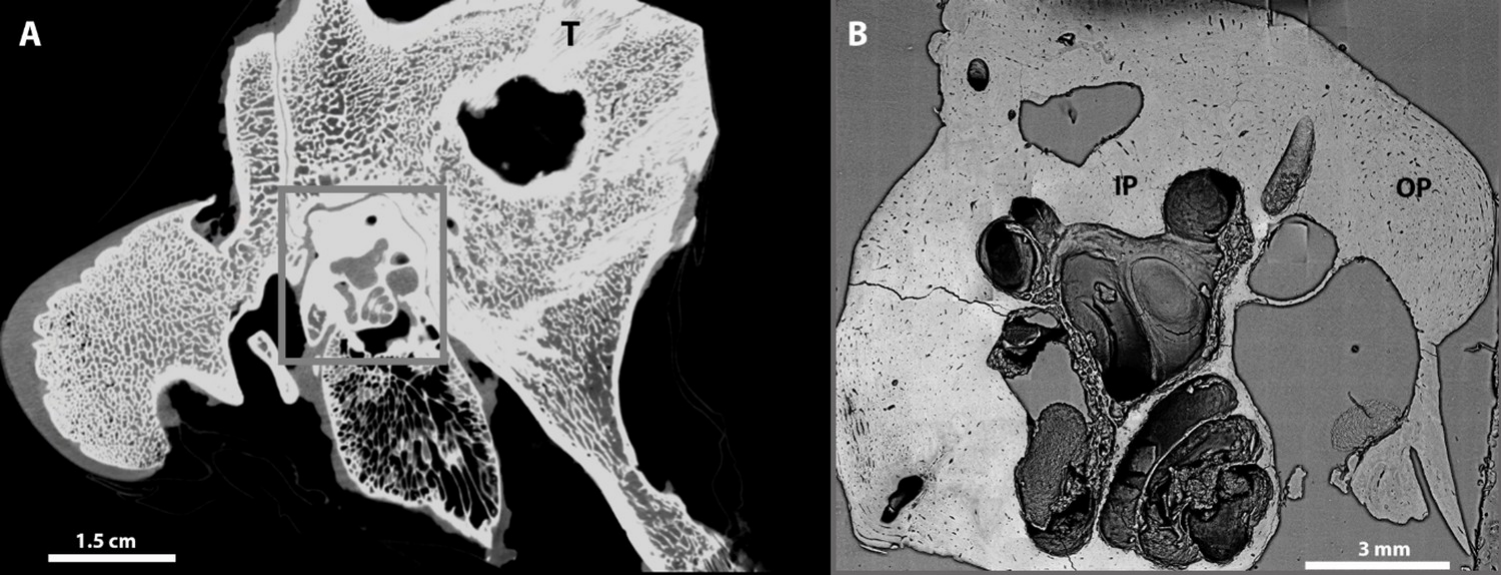
2D representation of the temporal bone including a longitudinal cross section of the otic capsule. (A) A slice from a low-resolution μ-CT tomogram obtained from the pig right temporal bone before extraction of the petrous bone showing the otic capsule in the grey rectangle. (B) SEM backscattered electron automated image map of a transverse section of a pig petrous bone after extraction, sectioning and polishing. The mastoid bone samples were collected from the same location (T) from two different pigs (Fig 2A). The locations of petrous bone samples from the outer and inner layers (OP and IP) are shown in Fig 2B. These samples were collected from the same locations in two different pigs.

The experimental strategy that we used was to compare lacunae concentrations in two of the regions of the petrous bone, one from the inner layer of the otic capsule (IP) (<1mm from the otic cavity) and one from the outer layer of the otic capsule (OP) (>3mm from the otic cavity). These layers were then compared to another bone in the skull complex (namely the mastoid part of the temporal bone). See Fig 2 for locations of IP, OP and T samples, and (S2 Movie in S1 File) showing a video of the petrous and mastoid bone complex. We also compared these bones to cortical bone from pig femora. Osteocyte lacunae concentrations have been studied in other animal femora [36, 37].

**Fig 3:**
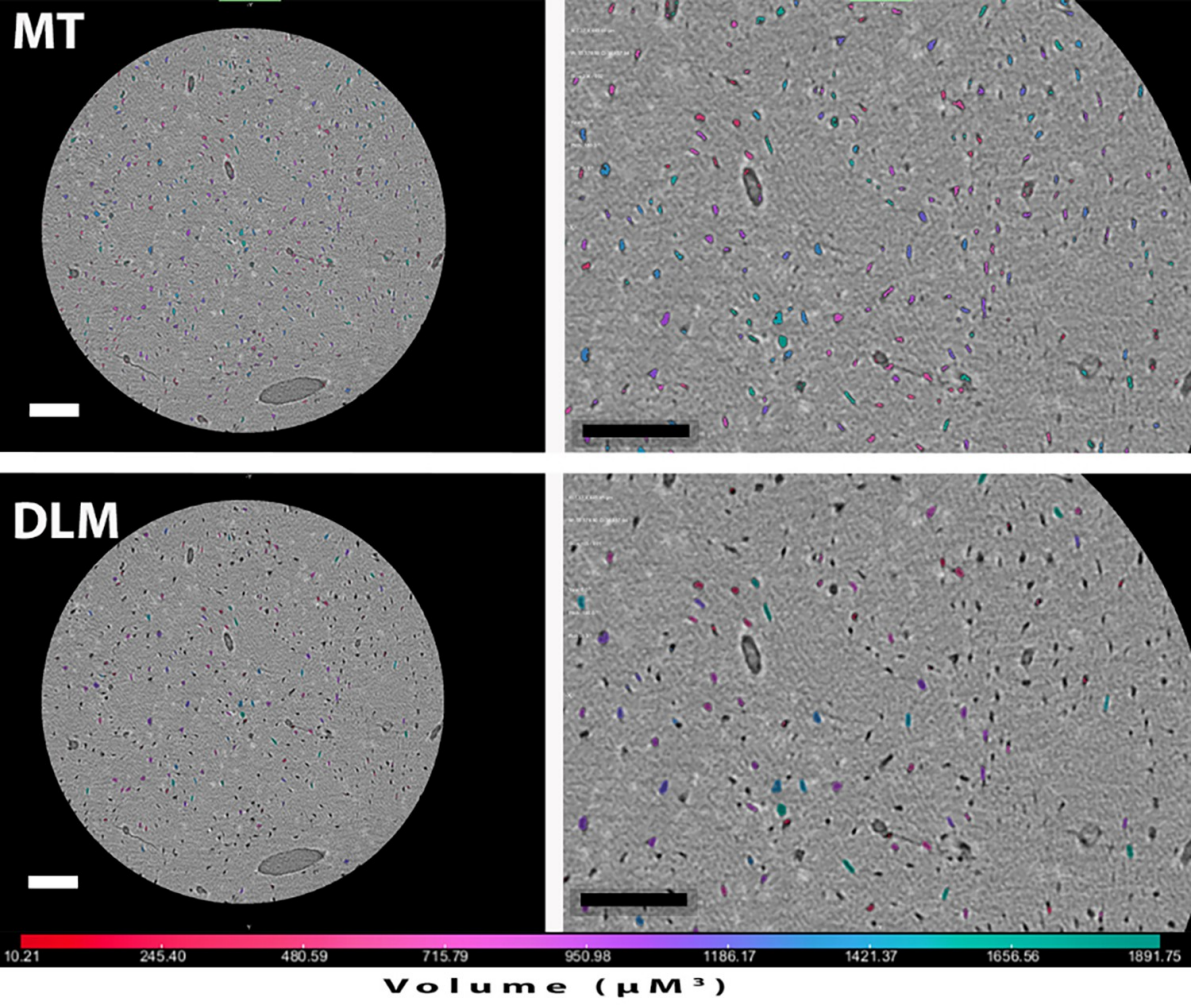
High resolution μ-CT tomogram slice of the inner layer sample (IP2) after MT (top) and DLM (bottom) segmentation. Left images show the whole field of view at the same slice location, and right images show insets of the OL labels as obtained from the two different segmentation approaches. Colors are a qualitative indication of the volume of the OL labels. Scale bars = 100μm.

**Fig 4:**
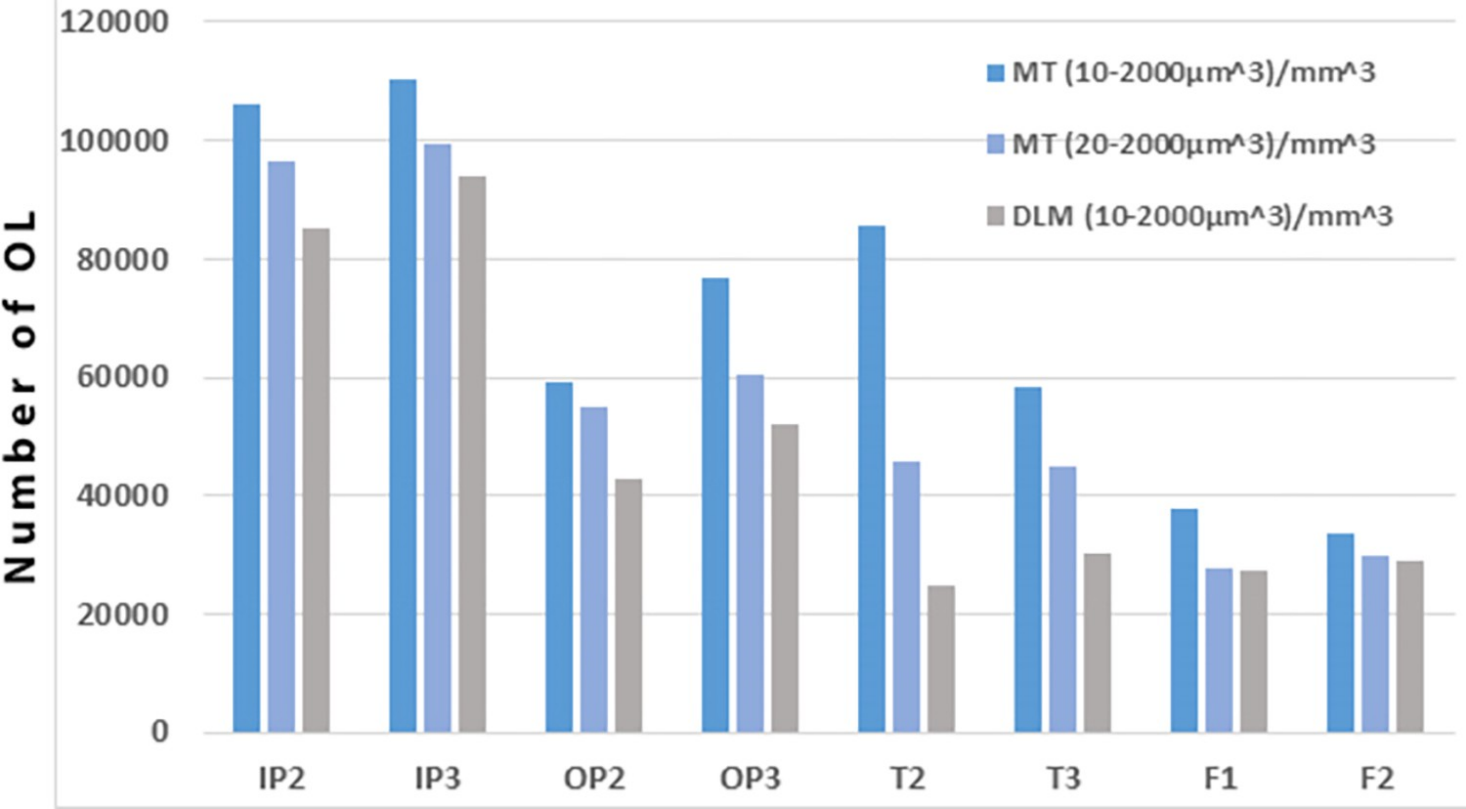
The OL concentration using the two segmentation approaches. Dark blue represents the MT segmentation with volumes from 10–2000μm^3^, light blue is the same segmentation method but with volumes from 20–2000 μm^3^, and grey represents the DLM segmentation for OL volumes between 10–2000μm^3^.

One high resolution micro-CT tomogram was obtained from each of the 8 samples: four from two petrous bones (two from the inner (IP) and two from the outer (OP) layers), two from mastoid bones (T) and two from femora (F)). The sub-micrometer voxel size of each μ-CT scan (Table 1) enabled the reliable detection of all osteocyte lacunae from all the different bone tissues [32, 38]. Osteocyte lacunae (OL) were counted and measured digitally from all sample scans following segmentation and analysis. Fig 3 shows low and high magnification views of a μ-CT slice from the inner petrous bone (IP) sample with the lacunae segmented according to the two methods (Mean Threshold (MT) and the Dragonfly deep-learning model (DLM)). Upon close examination of individual lacunae, it can be seen that the DLM segmentation labels cover a slightly larger area of OL than the MT segmentation labels (Fig 3). Table 1 shows the OL concentrations per mm^3^ of bone volume for both methods from all of the 8 samples. We used the same range of lacunae volumes that was used by [32], namely 10–2000 μm^3^, so that we could compare our results with theirs and by so doing validate the methods that we used for counting. However lacunae with volumes between 10–20 μm^3^ are very small and quite abundant. We therefore also used a range of 20–2000 μm^3^ for the MT method. The results are shown in Table 1 and plotted in Fig 4. Despite the different ranges and segmentation methods used, it is clear that both IP samples contain significantly more OL than the other bone samples. In fact the IP samples contain about 2-fold more OL than the OP samples, and about 3-fold more OL than the femora.

**Table 1:** Numbers of OL from all different samples.

Sample	Voxel size μm^3^	Scan Vol. mm^3^	MT (10–2000μm^3^)/mm^3^	MT (20–2000μm^3^)/mm^3^	DLM (10–2000μm^3^)/mm^3^
**IP2**	0.9	0.5	106000	97000	85000
**IP3**	0.9	0.6	110000	99000	94000
**OP2**	0.9	0.5	59000	55000	43000
**OP3**	0.8	0.4	77000	60000	52000
**T2**	0.8	0.4	85000	46000	25000
**T3**	0.9	0.5	58000	45000	30000
**F1**	0.8	0.3	38000	28000	27000
**F2**	0.8	0.3	34000	30000	29000

Table 1 shows the voxel size and scan volume (Vol.) for all μ-CT scans and the concentrations of OL from all samples. The mean threshold (MT) segmentation results are presented for OL concentrations in two OL volume ranges: one for OL volumes between 10–2000 μm^3^ -the range used by Carter et al. [32, 38]; and 20–2000 μm^3^. Table 1 shows one range for the deep-learning model (DLM) segmentation method for OL volumes between 10–2000 μm^3^.

**Fig 5:**
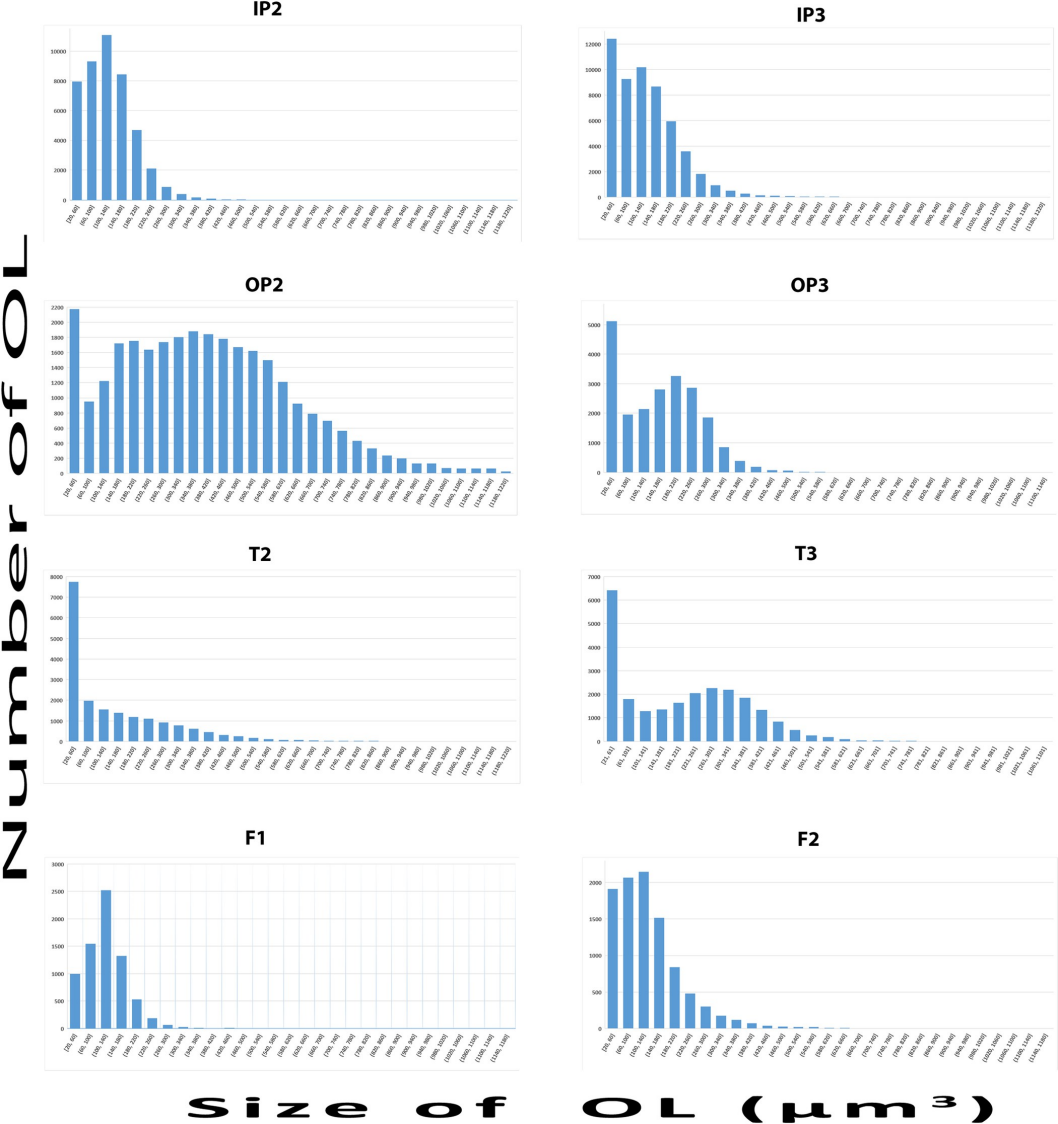
OL volume histograms from all the samples showing the OL volume distribution from 20 to 1200 μm^3^. OL volume average and standard deviations are provided in Table 2. Note that the unusual distribution of T2 may be due to the poor resolution of the scan (see Methods).

The next question we addressed was whether or not the OL in the 4 different bone samples have different volumes and/or shapes. Table 2 shows the average OL volumes and average aspect ratios obtained from the MT segmentation for labels between 10 and 2000 μm^3^. As the volume standard deviations are very large, we also plotted the distributions of OL as a function of volume (Fig 5). The OL in the inner capsular layer (IP) are smaller than those in the outer capsular layer (OP). The IP lacunae have similar volumes to those in the femora. The large standard deviation and the unusual distribution of T2 (Table 2), preclude concluding anything about its volume distribution. The aspect ratio values show no significant difference between all 8 samples.

**Table 2:** Morphometric analysis of lacunae from all samples.

Sample	Avg Vol. (μm^3^)	Vol. Stdev. (μm^3^)	Avg. AR	AR Stdev.
**IP2**	128	81	0.41	0.11
**IP3**	140	117	0.51	0.11
**OP2**	404	278	0.47	0.11
**OP3**	162	105	0.45	0.12
**T2**	166	190	0.49	0.11
**T3**	212	155	0.45	0.12
**F1**	122	72	0.34	0.10
**F2**	137	112	0.39	0.11

Table 2 shows OL average volume (Avg. Vol.) and average aspect ratio (Avg. AR) measurements of osteocyte lacunae segmented labels, together with their standard deviations (Stdev.) The data shown are obtained from the mean thresholding segmentation method (MT) for OL volume ranges between 20 and 2000 μm^3^.

**Fig 6:**
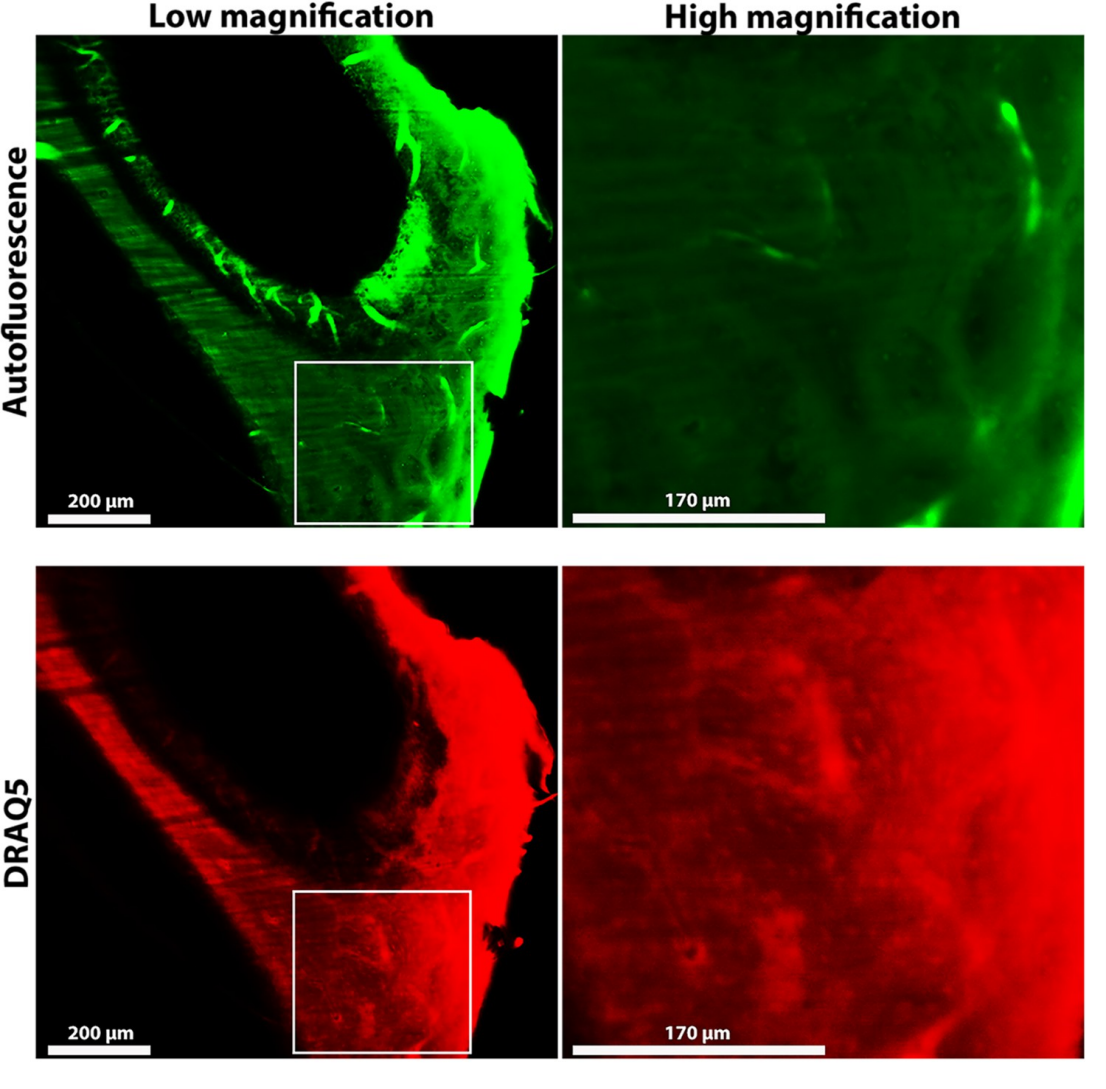
Light-sheet fluorescent microscopy images of a cleared pig petrous bone. All images are from the same Z-depth with (green) autofluorescence and (red) DRAQ5 signals. Low magnification images show the cleared bone tissue from the cochlear cavities towards the outer bone surface. High magnification images of areas indicated by the rectangles are shown. The local concentrations of DRAQ5 stained nuclei can best be seen in the high magnification image.

To check the viability of osteocytes in a section containing the outer and inner periosteal layers, we used DRAQ5 to stain the cell nuclei of a cleared and fluorescently dyed petrous bone from pig. As controls we also studied mouse bones as the method was developed using mouse bones (see S1 Fig in S1 File). The autofluorescence signal from the sample is thought to be derived mainly from collagen (green). Fig 6 shows light-sheet images obtained in two channels. The DRAQ5 nuclear staining shows the presence of nucleated osteocytes in the outer-inner periosteal bone layers outside the cochlea (red). The lacunae in the outer layers of the petrous bone therefore do contain osteocytes together with the DNA in their nuclei.

## Discussion

This study on pig shows that the inner layer (IP) has significantly more osteocyte lacunae than the outer layer of the petrous bone, and both these layers have more osteocyte lacunae than the femora. The mastoid of the temporal bone OL concentrations are less reliable but are certainly well below the concentration in the IP. Light-sheet fluorescence shows that the petrous bone contains concentrations of DNA inferred from signals similar to osteocytes in dimensions and distribution (See S3 Movie in S1 File). This conclusion is consistent with the quantitative observations of Bloch et al. [25] and the qualitative observation that the petrous bone is “highly osteocytic” [26, 27].

We can compare the OL concentrations and volumes that we obtained from the pig femora to concentrations reported in the long bones of other species. Carter et al. measured the OL concentrations in a 20 year old human male femur. The concentrations ranged between 26,000 and 37,000 per mm^3^ of bone [32]. Androwski et al. obtained 22,000 per mm^3^ from human cortical bone [29]. We obtained similar values from cortical pig femora. The lacunar concentrations reported by Bloch et al. for the inner and outer capsules of human petrous bones, namely 75–90,000 osteocytes per mm^3^ and 50–61,000 osteocytes per mm^3^ respectively [25], are similar to those reported here for the pig. The lacunar volumes measured in the human male are around 400 μm^3^, whereas in the human female group they are on average around 240 μm^3^ [32, 38]. Our measurements of the two pig femora OL volumes are on average 120 and 140 μm^3^ and are therefore considerably smaller than the human bone values reported by Carter et al. [32, 38].

These observations show that modern pig petrous bone, and in particular the part of the petrous bone near the otic chamber (IP layer), contains at least three times as many lacunae as the femoral bones of the pig. Thus the petrous bone of pigs and as reported in humans initially has much more DNA [25], and during fossilization (diagenesis) the petrous bone may also preserve more DNA. This therefore could be one reason why fossil petrous bones contain more aDNA than other bones [8, 9, 11]. It would therefore be interesting to carry out a systematic study of osteocyte lacunae concentrations in bones of the human skeleton, as other bones may also have high concentrations of lacunae and hence possibly high concentrations of aDNA in the same fossil bone. It would also be of interest to carry out a detailed 3D study of the structure of the petrous bone, as this may reveal other structural properties that might be responsible for the good preservation of aDNA. We are currently carrying out such a study using focused ion beam–scanning electron microscopy (FIB SEM).

It has been proposed that the higher mineral density of the petrous bone is responsible for the good preservation of aDNA [8]. It is known that DNA has a high affinity for carbonated apatite and that bound DNA is less susceptible to enzyme degradation [39, 40]. However higher bone mineral density results in a much lower surface area [41] and hence probably less DNA binding. It has also been suggested that the high mineral density of petrous bone reduces bacterial activity and therefore post-mortem decay of DNA [8]. It is not clear why higher mineral density per se should reduce bacterial activity. A reduction in bacterial activity may be the consequence of some unique structural features of the petrous bone, such as the characteristics of blood vessels and canaliculi. We do note that the petrous bone is reported to contain mineralized lacunae [15]. We however would not have detected these mineralized lacunae using microCT.

It was demonstrated that in the perilabyrinthine bone in-vivo inhibition of bone resorption preserves the fetal highly cellular primary bone [42, 43]. This inhibition is caused by expression of osteoprotegrin (OPG), a potent inhibitor of osteoclast formation and function. This mechanism assumes that OPG travels through intercellular gaps of the inner ear and diffuses to the capsular bone via the lacuna-canalicular network forming an “osseous functional network” [44]. It is interesting to consider the possible functions of having so many osteocytes in the petrous bone close to the otic chamber. Doden and Halves postulate that the otic capsule bone has a physiological function related to sensation [20]. Aarden et al. summarize the functions of osteocytes as follows: 1) communication bridges between inside and outside the bone tissue; 2) increasing the mineral surface exposed to extracellular fluid and cellular activity; 3) providing a repair mechanism in addition to remodeling [45]. While others show that osteocytes remodel bone matrix through perilacunar/canalicular remodeling (PLR) and they highlight the importance of osteocytes for improving bone fragility [46, 47]. The ability of osteocytes for mechanosensing is well documented [48]. Interestingly, Fung et al. exposed osteocytes to low intensity pulsed ultrasound (LIPUS). This stimulated the osteocytes and promoted the mechano-transduction between osteocytes and osteoblasts [49]. Furthermore, it has been proposed that OPG induced inhibition of remodeling close to the inner ear spaces preserves the electro-mechanical integrity of the inner ear. The high concentrations of osteocytes and their canaliculi result from the persistence of hypercellular fetal primary bone in this compartment [25, 50]. So when cells die and pericellular maintenance is stopped, the lacunae and canaliculi calcify and seal off apoptotic contents [51, 52], the high content of lacunae and canaliculi in bone close to the otic chamber may lead to higher preservation of endogenous DNA.

## Conclusions

We show that the petrous bone of the pig has three-fold more osteocyte lacunae, than the femoral compact bone and hence the pig petrous bone has significantly more DNA occluded inside the bone. This might be one factor that leads to the preservation of high amounts of DNA in fossil bones.

## Supporting information

S1 File (DOCX)
