# Supplementary material for: The petrous bone contains high concentrations of osteocytes: One possible reason why ancient DNA is better preserved in this bone
Source: PLoS One. 2022 Oct 25;17(10):e0269348. doi: 10.1371/journal.pone.0269348 (PMC9595551; doi:10.1371/journal.pone.0269348)
Supplement: S1 File — (DOCX) [file pone.0269348.s001.docx]

**Supporting Information:**

S1 Fig.


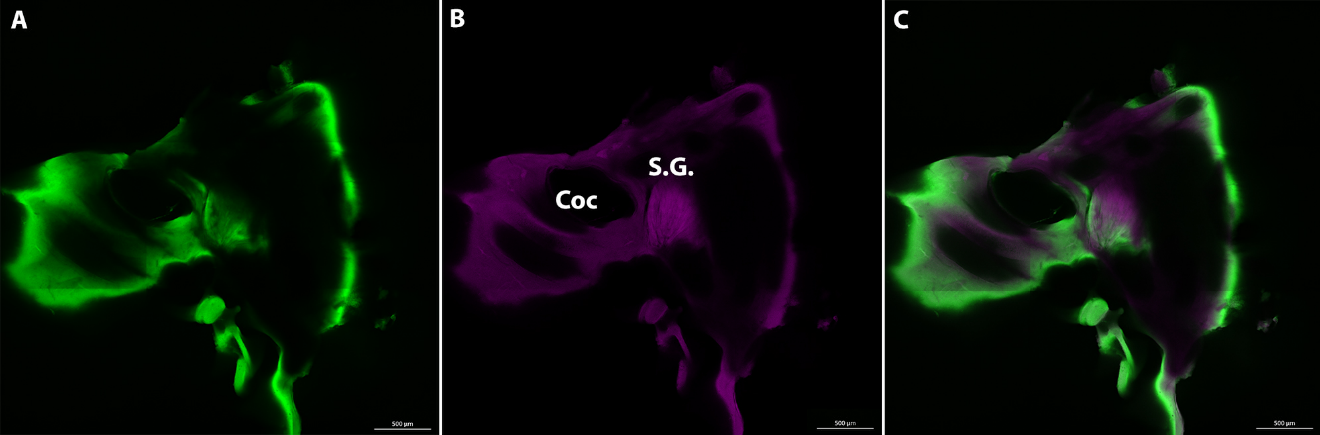


**S1 Fig:** **Fluorescent microscope images at the same Z-depth of mice petrous bone after total clearing using the PEGASOS protocol [33] and DNA staining using DRAQ5**. (A) Autofluorescence signal (green), (B) DRAQ5 signal (magenta), and (C) both signal channels combined. In (B and C) the DRAQ5 staining of the spiral ganglion (S.G.) and the inner membranes of the cochlea (Coc) clearly show high concentrations of DNA in the mouse petrous bone. This confirms that the protocol used for the pig petrous bone should be reliable, even though the pig petrous bone takes much longer time to clear. Scale bar = 500µm.

S2 Mov.

 **S2 Mov:** **Low resolution micro-CT scan of the extracted skull section from pig**. Showing the temporal bone including the petrous part, tympanic bullae, and zygomatic arch bones. Note that the petrous bone contains no spongey bone component. Pixel size = 83µm and scale bar = 1cm.

S3 Mov.

**S3 Mov:** **High resolution light-sheet 3D image of cleared petrous bone from pig. B** DNA stained using Draq5 is shown with a resolution of 2 micron approx. the stained osteocytes appear like spots distributed in high quantities in the bone surrounding the posterior cochlear walls.
